# Alterations in whole-brain white matter structural network among females with abdominal obesity by appetite subtypes

**DOI:** 10.3389/fnut.2026.1768938

**Published:** 2026-03-24

**Authors:** Siwen Zhao, Qifu Li, Gaoyangzi Huang, Wenjun Li, Yuanzheng Deng, Ruqin Yang, Shumin Zhang, Fanrong Liang, Yi Lu, Taipin Guo

**Affiliations:** 1 School of Second Clinical Medicine, The Second Affiliated Hospital, Yunnan University of Chinese Medicine, Kunming, Yunnan, China; 2 College of Acupuncture and Tuina, Chengdu University of Traditional Chinese Medicine, Chengdu, Sichuan, China; 3 The Department of Medical Imaging, The First Affiliated Hospital of Kunming Medical University, Kunming, Yunnan, China

**Keywords:** abdominal obesity, appetite subtypes, diffusion tensor imaging, graph theoretical, white matter

## Abstract

Abdominal obesity (AO) in women exhibits clinical heterogeneity, yet its neural mechanisms remain unclear. Using diffusion tensor imaging (DTI), this study conducts the first systematic investigation of whole-brain white matter (WM) structural network topological properties and female-specific neural mechanisms in AO patients stratified by appetite subtypes (strong appetite, SA; moderate appetite, MA). This study enrolled 60 female AO patients (stratified into 30 SA and 30 MA) and 30 age-matched healthy controls (HCs). Whole-brain WM structural networks were constructed, with global and local topological properties analyzed through graph theoretical approaches. Statistical correlations between network metrics and clinical measures were assessed. Global network analysis demonstrated intact small-world properties (*γ* > 1, *σ* > 1) across all groups. At the nodal level, compared with HCs, SA patients showed reduced degree centrality (DC) in the right temporal pole: superior temporal gyrus (TPOsup. R) (*p* = 0.017) and lower nodal local efficiency (NLE) in the right amygdala (AMYG. R) (*p* = 0.007). Notably, TPOsup. R DC positively correlated with appetite scores (*r* = 0.408, *p* = 0.025). Additionally, the SA group exhibited reduced betweenness centrality (BC) in the right rectus gyrus (REC. R) compared to the MA group (*p* = 0.041), which negatively correlated with appetite scores (*r* = −0.439, *p* = 0.015). Conversely, MA patients showed increased NLE in the right medial orbital superior frontal gyrus (ORBsupmed. R) (*p* = 0.005). Whole-brain connectome analysis identified a hypo-connected subnetwork specific to MA involving 23 nodes and 24 edges, highlighting weakened right amygdala (AMYG. R)–right superior occipital gyrus (SOG. R) (*r* = 0.464, *p* = 0.01) and right lingual gyrus (LING. R)–right fusiform gyrus (FFG. R) (*r* = 0.365, *p* = 0.047) connections that showed positive correlation with waist circumference. In summary, this study provides the first evidence that female patients with abdominal obesity exhibiting different appetite phenotypes possess specific white matter structural network patterns. The SA subtype is characterized by aberrant nodal topological properties within prefrontal-limbic circuits, reflecting a localized disruption associated with heightened appetite. In contrast, the MA subtype is defined by widespread structural disconnectivity between occipital sensory areas and limbic regions, appearing as a neuroanatomical consequence linked to abdominal adiposity severity.

## 1 Introduction

Abdominal obesity (AO), the core phenotype of metabolic syndrome characterized by abnormal visceral adipose tissue accumulation, carries significantly greater pathological risks than generalized obesity (1, 2). Epidemiological surveys have shown that the rate of AO among middle-aged women is 34.08%, which is substantially higher than the rate of 31.99% in men in China (3). This rate has increased from 19.84% to 43.15% over 20 years, making it the primary metabolic disease threatening women’s health (4). In addition to its known associations with cardiovascular diseases and type 2 diabetes, female with AO significantly increase the risks of polycystic ovary syndrome, reproductive dysfunction, and estrogen-dependent malignancies (5, 6). These sex-specific risks are closely related to the distinct hormonal fluctuation patterns, fat metabolism regulatory mechanisms, and visceral fat distribution characteristics in women (7). Research has shown that estrogen can directly regulate the dynamic balance of visceral fat metabolism in women by regulating neuronal activity in the ventromedial hypothalamic nucleus (8).

Appetite regulation is the central axis of energy homeostasis. It involves dynamic integration within the hypothalamic-nucleus accumbens dopamine circuitry. Recent research has confirmed that dopamine receptor 1 expressing medium spiny neurons in the nucleus accumbens inhibit eating through leptin receptors projected from the lateral hypothalamus (9). However, “hedonic hunger” is common in females with AO, which refers to a persistent craving for high-calorie foods despite sufficient energy intake (10). Abnormal functioning of the neurotensin neurons in the lateral septal nucleus has been suggested to lead to such pathological eating behaviors (11). Notably, our preliminary clinical trials found that the same intervention method has significantly better clinical efficacy in females with AO and a strong appetite (SA) than in those with a moderate appetite (MA) (12, 13), suggesting that different appetite subtypes may correspond to differentiated neural regulation mechanisms. This raises a critical scientific question: Do females with AO different appetite subtypes have specific reorganization patterns in their brain structural networks?

Previous diffusion tensor imaging (DTI) studies have primarily examined region-specific white matter (WM) microstructural alterations in obese populations, such as reduced fractional anisotropy (FA) and increased axial diffusivity (AD) within frontal-limbic fiber tracts (14–16), however, few studies have analyzed the overall pattern of structural connections at the level of whole-brain network topological attributes. Structural network analysis has found that the degree centrality (DC) of WM fiber bundles such as the cerebral peduncle and corona radiata is abnormal in female with AO and is associated with eating behavior dysregulation (17), however, it has not been compared in a hierarchical manner within the framework of appetite typing, making it difficult to explain the clinical heterogeneity of SA and MA patients. A recent multimodal study suggested that diminished effective connectivity between the executive control network and reward network may be a neural biomarker for binge eating behavior (18). In particular, abnormal AD alterations have been identified along front-limbic-parietal pathways in women with appetite dysregulation-related obesity (19). These findings indicate that different appetite subtypes may impact treatment response by leading to specific WM network reorganization (e.g., differences in the topological properties of the prefrontal-striatal pathway), with sex differences also being a factor. However, this hypothesis requires further verification through a systematic analysis of the structural network.

Graph theory and network-based statistics (NBS) analysis are two commonly used methods in whole-brain network analysis. They can construct a network of brain structures in obese patients, analyze the connections in the structural network, and further elucidate the pathogenesis of obesity (20, 21). Based on this, this study combined graph theory analysis with the NBS method for the first time to construct a 90-node whole-brain WM structural network and systematically explore the differences in global topological properties and subnetwork connection strengths among SA, MA, and HC. The following questions were addressed: (1) Do SA and MA patients have characteristic differences in global topological properties? (2) How is the structural connectivity strength of key subnetworks (e.g., dorsolateral prefrontal cortex-striatal pathway) dynamically associated with clinical phenotypes such as appetite scores and waist circumference?

## 2 Materials and methods

### 2.1 Study design

**Figure 1:**
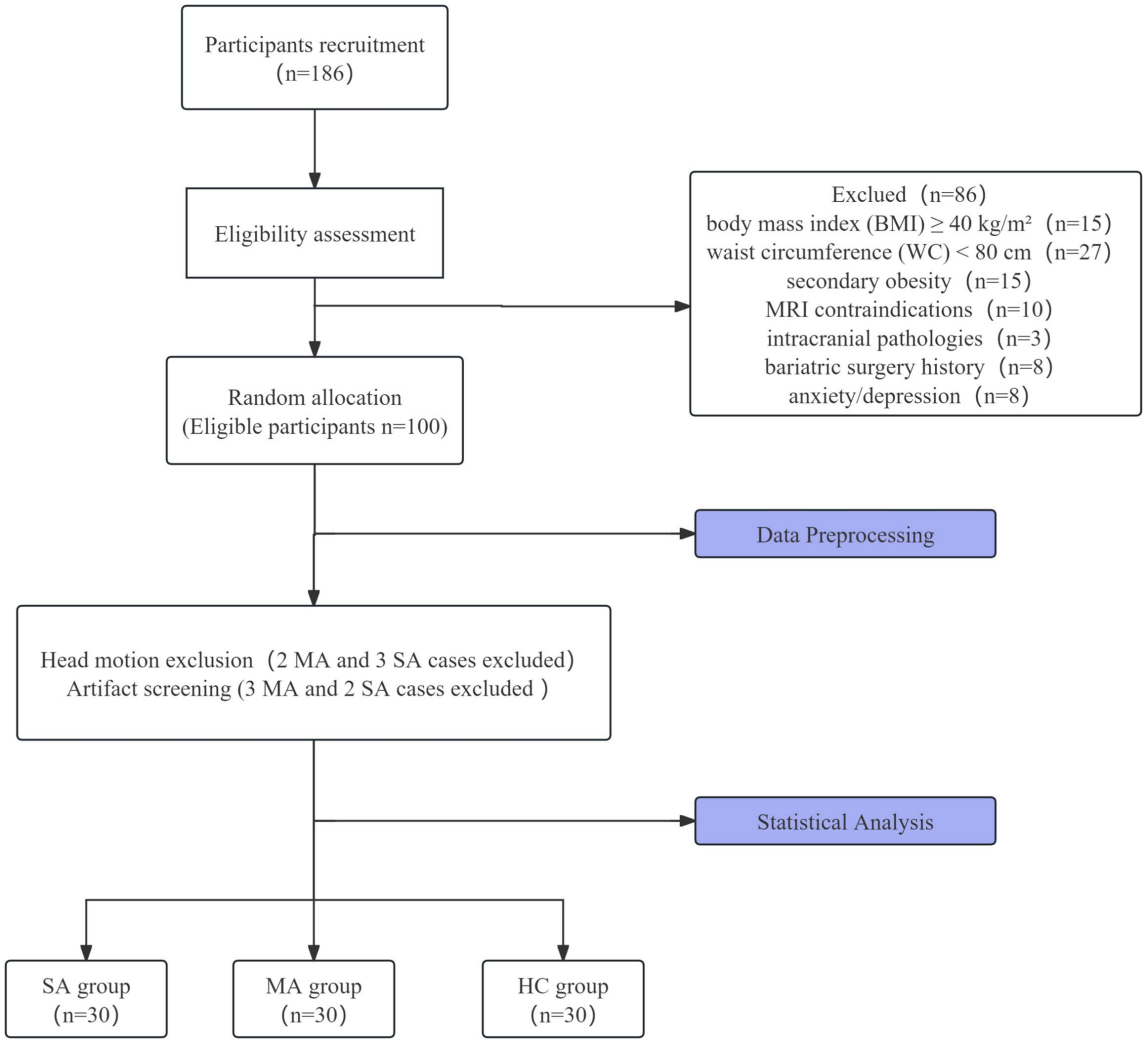
Flow chart of participants’ inclusion and exclusion process.

This cross-sectional study used baseline data from a registered clinical neuroimaging trial (ChiCTR2100048920) (22). A total of 70 women with AO and 30 HCs were recruited from March 2021 to May 2024. All participants provided written informed consent. The study protocol was approved by the Medical Ethics Committee of Yunnan Provincial Sports Trauma Specialist Hospital (Approval No. 2021–01) and was conducted in accordance with the Declaration of Helsinki. DTI analyses were performed to analyze between-group differences in whole-brain WM structural network topology among the MA, SA, and HC groups. The research flowchart is shown in Figure 1.

### 2.2 Participants

The study enrolled females with AO patients meeting the diagnostic criteria based on the guidelines established by the 2011 Chinese Expert Consensus on the Prevention and Treatment of Adult Obesity (23), which included a body mass index (BMI) of ≥28 kg/m^2^ and a waist circumference (WC) of ≥80 cm. During recruitment, we strictly controlled the participants’ hunger state. Following a standardized overnight fast (of at least 8 h), appetite subtypes were assessed using a Visual Analogue Scale (VAS) (0–10 points) (24), classifying participants into the SA group (scores 7–10) and the MA group (scores 4–6). These stratification criteria were established based on our team’s prior clinical trials (12, 13). Specifically, participants stratified by these cut-offs exhibited significant clinical differences under the same intervention protocols, thereby validating the rationale for this classification. The inclusion criteria were as follows: right-handedness, age 18–40 years, and ≥6 years of education. The exclusion criteria were as follows: BMI ≥ 40 kg/m^2^, secondary obesity (e.g., hypothalamic lesions, Cushing’s syndrome, hypothyroidism, drug-induced obesity), pregnancy/lactation, major comorbidities (e.g., diabetes, cardiovascular, psychiatric, immunodeficiency, organ failure diseases), magnetic resonance imaging (MRI) contraindications (e.g., metallic implants, pacemakers, claustrophobia), intracranial pathologies, bariatric surgery history, and anxiety/depression: Self-Rating Depression Scale score ≥ 53. HCs were age- and education-matched right-handed females with a BMI of 18.5–23.9 kg/m^2^ and WC of <80 cm, subjected to identical exclusion criteria.

### 2.3 MRI acquisition

All participants underwent MRI data acquisition using a GE Discovery 750 W 3 T MRI system (16-channel head coil). Prior to scanning, participants removed all ferromagnetic objects and were positioned supine with head immobilization using compressible foam pads thermoplastic masks, and earplugs for noise reduction. During scanning, participants remained awake, eyes closed, breathing naturally, and refrained from structured cognitive activities. High-resolution 3D T1-weighted structural imaging with a fast spoiled gradient echo (BRAVO) sequence was conducted with the following parameters: repetition time (TR) = 7.7 ms, echo time (TE) = 3.6 ms, matrix = 228 × 228, and field of view (FOV) = 250 × 250 mm^2^, 230 axial slices. DTI images were acquired using an echo planar imaging (EPI) sequence with the following parameters: TR = 7,173 ms, TE = 78 ms, matrix = 115 × 115, FOV = 230 × 230 mm^2^, 50 contiguous axial slices (3 mm thickness), *b*-value = 1,000 s/mm^2^, and 32 diffusion directions. T2-weighted imaging was conducted as follows: TR = 2,500 ms, TE = 80 ms, matrix = 332 × 225, FOV = 250 × 220 mm^2^, and 18 axial slices (6 mm thickness). Quality assurance measures comprised real-time monitoring with scan termination/rescheduling for severe discomfort (e.g., claustrophobia, positional pain); head motion exclusion (>2 mm translation or >2° rotation.

### 2.4 Structural brain network construction

This study constructed whole-brain WM structural networks using MATLAB and the open-source PANDA toolbox (25). The specific workflow was as follows:

DTI data processing: This included skull stripping, eddy current and head motion correction, FA value calculation, and whole-brain deterministic DTI fiber tractography (26). First, non-brain tissues were removed from the raw DTI data using the built-in BET tool [parameters: intensity threshold 0.5, vertical crop 50 mm, gradient weight (*g*) = 0.2] (27). Next, head motion (6 degrees-of-freedom rigid registration) and eddy current distortions (nonlinear correction based on gradient direction tables) in diffusion-weighted images were jointly corrected via linear affine transformation (28). The diffusion tensor matrix was estimated using weighted linear least squares to compute whole-brain FA. Fiber tractography was performed using the FACT algorithm, with seeding from WM voxels with FA > 0.2. Tracking termination criteria included FA < 0.2, fiber turning angle > 45°or exceeding brain tissue boundaries, and retaining only fiber tracts ≥20 mm in length.

Network node definition: The AAL-90 template was adopted (29). Individual T1-weighted images were registered to native DTI b0 images using the FLIRT tool, followed by nonlinear normalization to MNI-152 space via FNIRT. Finally, the AAL template was inversely mapped to individual DTI space, partitioning 90 brain regions (45 per hemisphere) (30).

Structural connectivity matrix: Edge weights were defined as the average FA value along fiber tracts connecting two brain regions, retaining only edges with ≥ 1 fiber tract (FN ≥ 1). This threshold was selected to maintain a balance between sensitivity and specificity, preventing the exclusion of genuine but anatomically thin long-range association fibers. Furthermore, potential false-positive connections were mitigated by the subsequent sparsity-based thresholding in the network analysis, which naturally filters out weak or spurious edges. A symmetrical 90 × 90 weighted matrix was constructed and normalized to the [0, 1] range. Quality control included the exclusion of data with excessive head motion (translation > 2 mm or rotation > 2°), verification of FA distribution consistency through Pearson’s correlation coefficient (*r* > 0.85), and manual inspection of 10% randomly selected fiber tractography results. After excluding five MA and five SA participants due to excessive head motion and image artifacts (quality index < 0.8), the final analytic sample comprised 30 MA, 30 SA, and 30 HC participants.

### 2.5 Graph-theoretical analysis of structural brain networks

Using the GRETNA toolbox, this study employed graph-theoretical analyses to systematically assess topological differences in whole-brain WM structural networks among the three groups (SA, MA, and HC) (31). Network connectivity matrices were normalized via a sparsity thresholding approach, retaining each participant’s top S% strongest connections (sparsity range: 5%–40%, step = 1%) to ensure cross-group consistency in node count and connection density. This threshold range was selected to ensure small-world attributes were maintained while minimizing spurious edges, consistent with previous methodologies (31–33). Within this threshold range, global and local topological metrics were calculated (34), including global properties (small-worldness *σ*; normalized characteristic path length *λ*; normalized clustering coefficient *γ*; characteristic path length L_p_; clustering coefficient C_p_; local efficiency E_local_; global efficiency E_global_) and nodal topological properties (degree centrality, DC; nodal efficiency, NE; betweenness centrality, BC; nodal local efficiency, NLE). All metrics were computed as area-under-the-curve (AUC) values across the sparsity range for subsequent statistical analyses to mitigate single-threshold bias. The group differences in AUC values of global network metrics and nodal properties were investigated with one-way ANOVA, as preliminary demographic analyses showed no significant differences in age or disease duration among the groups. Metrics showing a main effect of group differences were further evaluated by post-hoc tests. A significance threshold of *p* < 0.05 was applied, and the false discovery rate (FDR) correction was performed to adjust for multiple comparisons across global and nodal metrics separately. To validate the robustness of the findings and disentangle the specific effects of appetite subtypes from obesity severity, sensitivity analyses were performed using General Linear Models exclusively within the AO cohort (comparing SA *vs.* MA). In these models, BMI and WC were included as covariates to isolate appetite-related independent effects, while avoiding the multicollinearity and over-adjustment bias introduced by the inclusion of healthy controls. Network visualizations were generated using BrainNet Viewer (35).

### 2.6 NBS analysis

To identify significantly altered subnetwork connections among the SA, MA, and HC groups, NBS analysis (http://www.nitrc.org/projects/nbs) was performed (36). The procedure involved four steps: 1) Supra-threshold link formation: One-tailed t-tests were conducted for each AAL-90 regional pair to compute FA-weighted connectivity strength differences (threshold: *t* = 2.5, degrees of freedom = 58, *p* < 0.05); 2) Subnetwork extraction: Topologically contiguous clusters (i.e., interconnected edge sets) were identified among supra-threshold links; 3) Permutation testing: Nonparametric permutation (5,000 iterations) was used to calculate family-wise error-corrected *p*-values for each subnetwork, retaining those with corrected *p* < 0.05; and 4) Visualization: Significant subnetworks were visualized using BrainNet Viewer.

### 2.7 Correlations between network measures and clinical variables

For nodes/edges showing significant between-group differences in graph-theoretical or NBS analyses, Pearson’s correlation analysis was conducted to investigate the associations with appetite subtype (appetite scores) and clinical indices of AO (BMI; body weight, BW; WC; disease duration). Statistical analyses were performed using IBM SPSS Statistics (Version 27.0; IBM, Armonk, NY, United States), controlling for age as a confounding factor, with statistical significance set at *p* < 0.05 (two-tailed tests).

## 3 Results

### 3.1 Demographics and clinical characteristics

The demographics and clinical characteristics of all participants are presented in Table 1. No significant differences were observed among the three groups in terms of age or the duration of obesity, along with between the MA and SA groups (*p* > 0.05). However, significant differences were detected in clinical features, including BMI, WC, BW, and appetite scores (*p* < 0.001). *Post hoc* comparisons revealed that both the SA and MA groups exhibited significantly higher BW, BMI, and WC than the HC group (*p* < 0.001), with the SA group showing markedly elevated BW, BMI, and WC values compared with the MA group (*p* < 0.001). Additionally, appetite scores in the SA group were significantly higher than those in the other groups (*p* < 0.001).

**Table 1:** Demographic and clinical characteristics and global topological properties of the SA, MA, and HC groups.

Characteristics	SA (*n* = 30)	MA (*n* = 30)	HC (*n* = 30)	F/*χ*^2^	*p*	*p*-value (SA *vs.* MA)	*p*-value (SA *vs.* HC)	*p*-value (MA *vs.* HC)
Age (y)	29.06 ± 0.96	31.90 ± 1.21	32.20 ± 1.24	2.254	0.111	0.256	0.173	1.000
Duration of disease (y)	6.63 ± 0.58	6.84 ± 0.89	—	0.038	0.847	0.847	—	—
BW	77.50 ± 1.36	74.08 ± 0.92	61.94 ± 1.99	29.205	<0.001	<0.001	<0.001	<0.001
BMI	30.55 ± 0.47	29.43 ± 0.24	21.21 ± 0.85	26.205	<0.001	<0.001	<0.001	<0.001
WC	101.35 ± 1.53	95.36 ± 1.08	84.48 ± 2.19	33.894	<0.001	<0.001	<0.001	<0.001
Appetite VAS score	8.30 ± 0.11	5.36 ± 0.11	5.13 ± 0.14	199.55	<0.001	<0.001	<0.001	0.570
σ	1.274 ± 0.013	1.273 ± 0.015	1.279 ± 0.018	0.042	0.944	0.971	0.817	0.789
*λ*	0.488 ± 0.001	0.489 ± 0.001	0.489 ± 0.001	0.258	0.817	0.563	0.513	0.940
*γ*	1.382 ± 0.017	1.383 ± 0.017	1.391 ± 0.023	0.060	0.922	0.980	0.759	0.776
C_p_	0.187 ± 0.001	0.184 ± 0.001	0.188 ± 0.002	1.826	0.178	0.161	0.684	0.072
L_p_	1.001 ± 0.005	1.007 ± 0.005	1.004 ± 0.003	0.600	0.576	0.306	0.404	0.849
E_glob_	0.205 ± 0.001	0.203 ± 0.001	0.204 ± 0.001	0.700	0.581	0.244	0.472	0.653
E_loc_	0.280 ± 0.002	0.280 ± 0.001	0.282 ± 0.002	1.316	0.271	0.526	0.333	0.111

BW, body weight; WC, waist circumference; BMI, body mass index; σ, small-worldness; *λ*, the normalized characteristic path length; *γ*, the normalized clustering coefficient; Cp, the clustering coefficient; Lp, the characteristic path length; Eglob, the global efficiency; Eloc, the local efficiency; SA, strong appetite; MA, moderate appetite; HC, healthy control.

### 3.2 Analysis of global topological properties in structural networks

**Figure 2:**
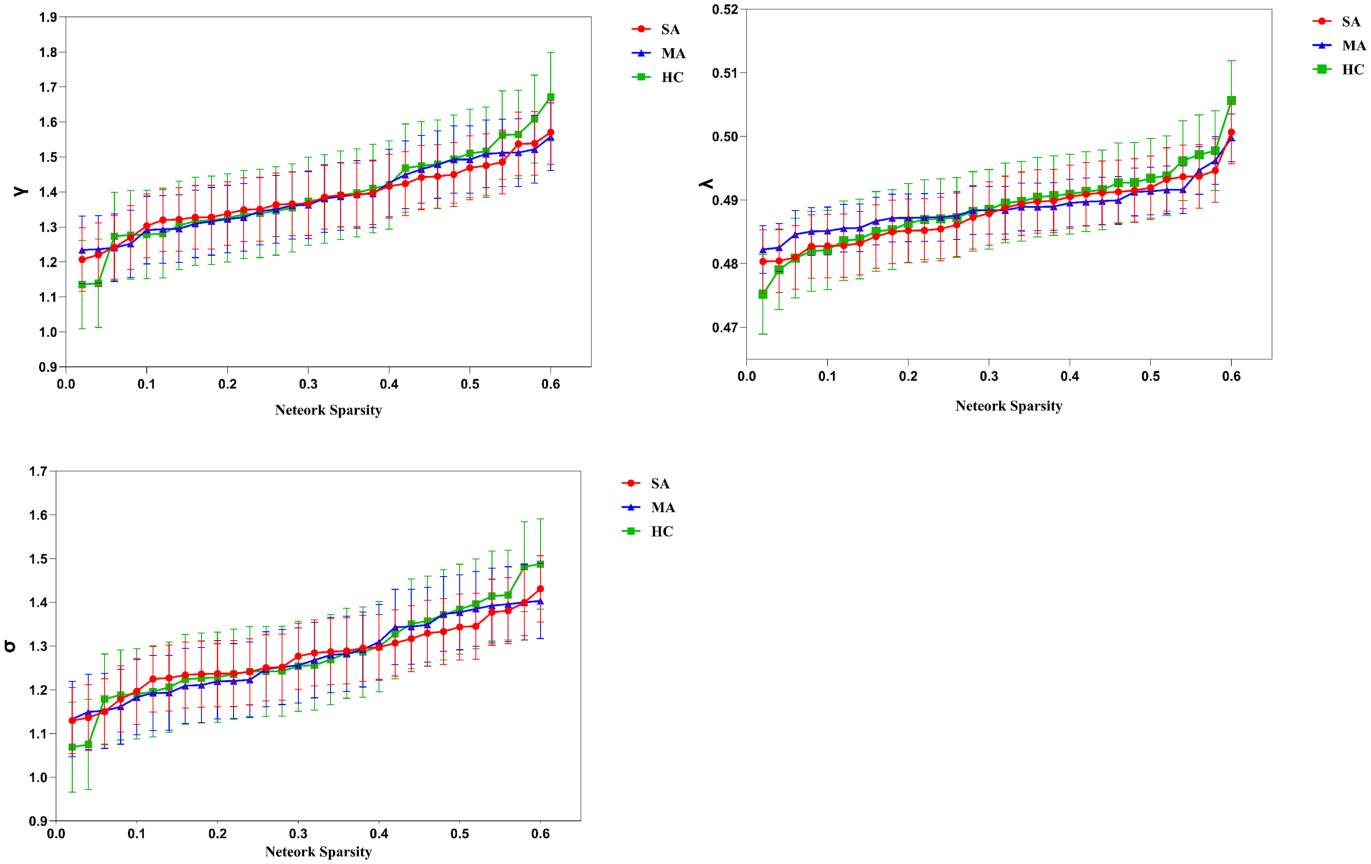
Results of small-world properties of the three groups under different sparsity. Bars and error bars represent mean values and standard deviation, respectively. *λ*, Normalized characteristic path length; *γ*, normalized clustering coefficient; *σ*, small-worldness; SA, strong appetite; MA, moderate appetite; HC, healthy control.

In the global network architecture, all three groups demonstrated intact small-world topology (*γ* > 1, *λ* ≈ 1, *σ* > 1; Figure 2), with no significant intergroup differences in small-world parameters (*p* > 0.05). Furthermore, there were no significant differences in the other global network metrics, including γ, λ, C_p_, L_p_, E_glob,_ and E_loc_, among the groups (*p* > 0.05, Table 1).

### 3.3 Analysis of nodal topological properties

**Figure 3:**
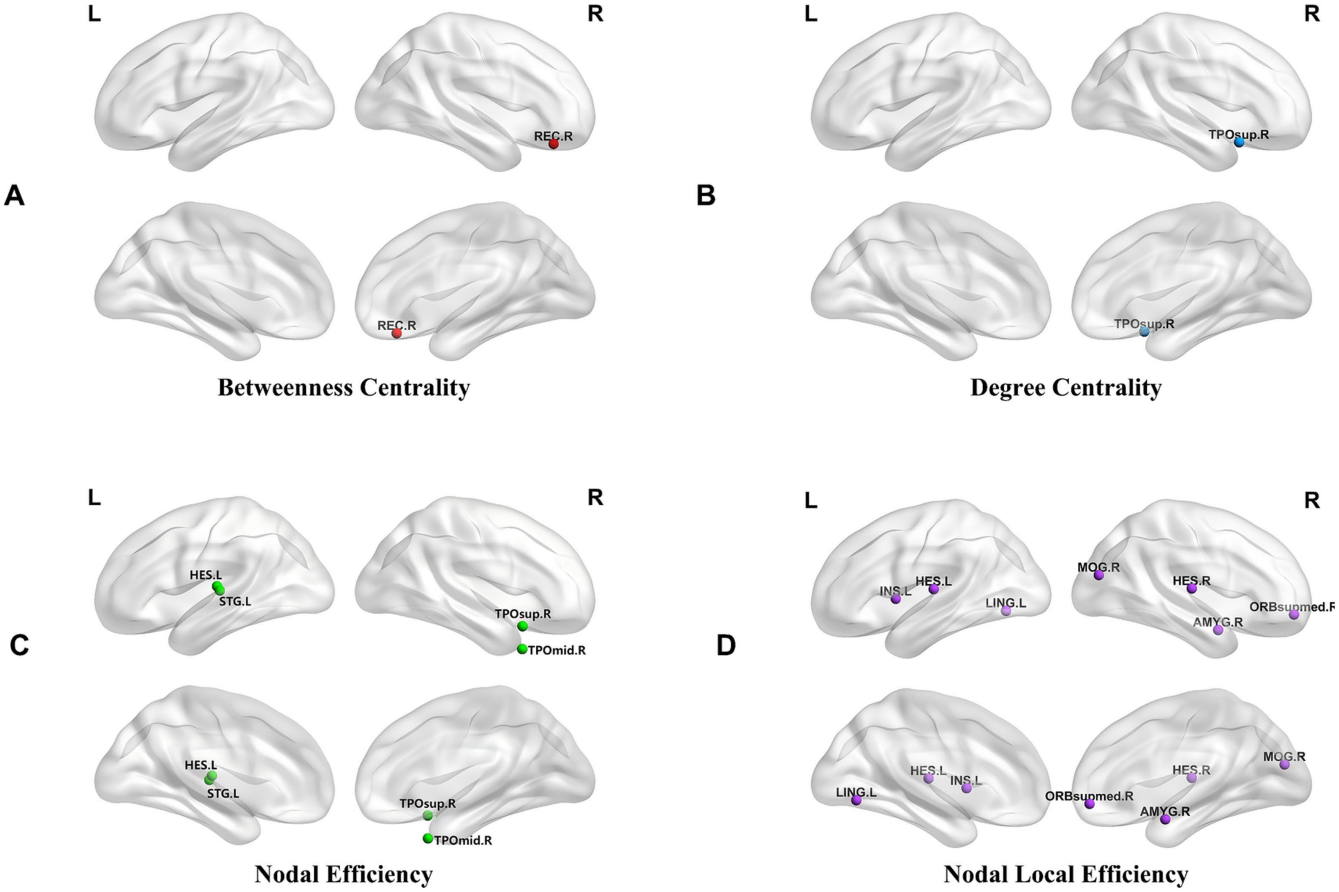
Group-level comparison of nodal properties. The distribution of differences in node characteristics among the three groups was determined using one-way ANOVA. **(A)** Betweenness centrality property; **(B)** degree centrality property; **(C)** nodal efficiency property; **(D)** nodal local efficiency property. The significance threshold was set at *p* < 0.05 (FDR corrected). REC. R, right rectus gyrus; TPOsup. R, right temporal pole: superior temporal gyrus; HES. L, left Heschl gyrus; STG. L, left superior temporal gyrus; TPOmid. R, right temporal pole: middle temporal gyrus; INS. L, left insula; LING. L, left lingual gyrus; MOG. R, right middle occipital gyrus; HES. R, right Heschl gyrus; AMYG. R, right amygdala; ORBsupmed. R, right medial orbital superior frontal gyrus.

As shown in Table 2 and Figure 3, significant differences in nodal topological properties were detected across multiple brain regions among the groups (FDR corrected, *p* < 0.05). Specifically, the SA group exhibited lower BC in the right rectus gyrus (REC. R) than the MA group (*p* = 0.041), whereas both the SA and MA groups showed reduced DC in the right temporal pole: superior temporal gyrus (TPOsup. R) compared with the HC group (SA vs. HC: *p* = 0.017; MA *vs.* HC: *p* = 0.018). For NE, the SA group demonstrated higher values in the left superior temporal gyrus than the MA group (*p* = 0.020), whereas the MA group had decreased NE in the TPOsup. R (*p* = 0.024) and right middle temporal gyrus (*p* = 0.044) compared with the HC. Analyses of NLE revealed that the SA group had reduced values in the left insula (INS. L) (*p* = 0.006) and right amygdala (AMYG. R) (*p* = 0.007), whereas the MA group exhibited lower NLE in the left lingual gyrus (LING. L) (*p* = 0.049) and left Heschl gyrus (*p* = 0.034) but elevated NLE in the right medial orbital superior frontal gyrus (ORBsupmed. R) (*p* = 0.005) compared with the HC group. Notably, Ne in the left Heschl gyrus and NLE in the right Heschl gyrus did not survive FDR correction (*p* > 0.05). Furthermore, sensitivity analysis restricted to the AO cohort confirmed that after controlling for BMI and WC, the nodal differences between the SA and MA subtypes (e.g., in STG. L, MOG. R, and REC. R) remained significant (Supplementary Table S1).

**Table 2:** Differences in the nodal topological properties among the SA, MA, and HC groups.

Nodes	ANOVA	*Post-hoc* test (*p*-value)			Difference
	(*p*-value)	SA *vs.* MA	SA *vs.* HC	MA *vs.* HC	
Degree centrality					
TPOsup. R	0.012	ns	0.017	0.018	SA, MA < HC
Nodal efficiency					
HES. L	0.048	ns	ns	ns	ns
STG. L	0.022	0.020	ns	ns	MA < SA
TPOsup. R	0.016	ns	ns	0.024	MA < HC
TPOmid. R	0.041	ns	ns	0.044	MA < HC
Nodal local efficiency					
ORBsupmed. R	0.005	ns	ns	0.005	MA > HC
INS. L	0.009	ns	0.006	ns	SA < HC
AMYG. R	0.003	ns	0.007	ns	SA < HC
LING. L	0.041	ns	ns	0.049	MA < HC
MOG. R	0.036	0.045	ns	ns	MA < SA
HES. L	0.023	ns	ns	0.034	MA < HC
HES. R	0.028	ns	ns	ns	ns
Betweenness centrality					
REC. R	0.021	0.041	ns	ns	SA < MA

The significance threshold was set at *p* < 0.05 (FDR corrected). SA, strong appetite; MA, moderate appetite; HC, healthy control; ns, non significance; REC. R, right rectus gyrus; TPOsup. R, right temporal pole: superior temporal gyrus; HES. L, left Heschl gyrus; STG. L, left superior temporal gyrus; TPOmid. R, right temporal pole: middle temporal gyrus; INS. L, left insula; LING. L, left lingual gyrus; MOG. R, right middle occipital gyrus; HES. R, right Heschl gyrus; AMYG. R, right amygdala; ORBsupmed. R, right medial orbital superior frontal gyrus.

### 3.4 Network-based statistical analysis

**Figure 4:**
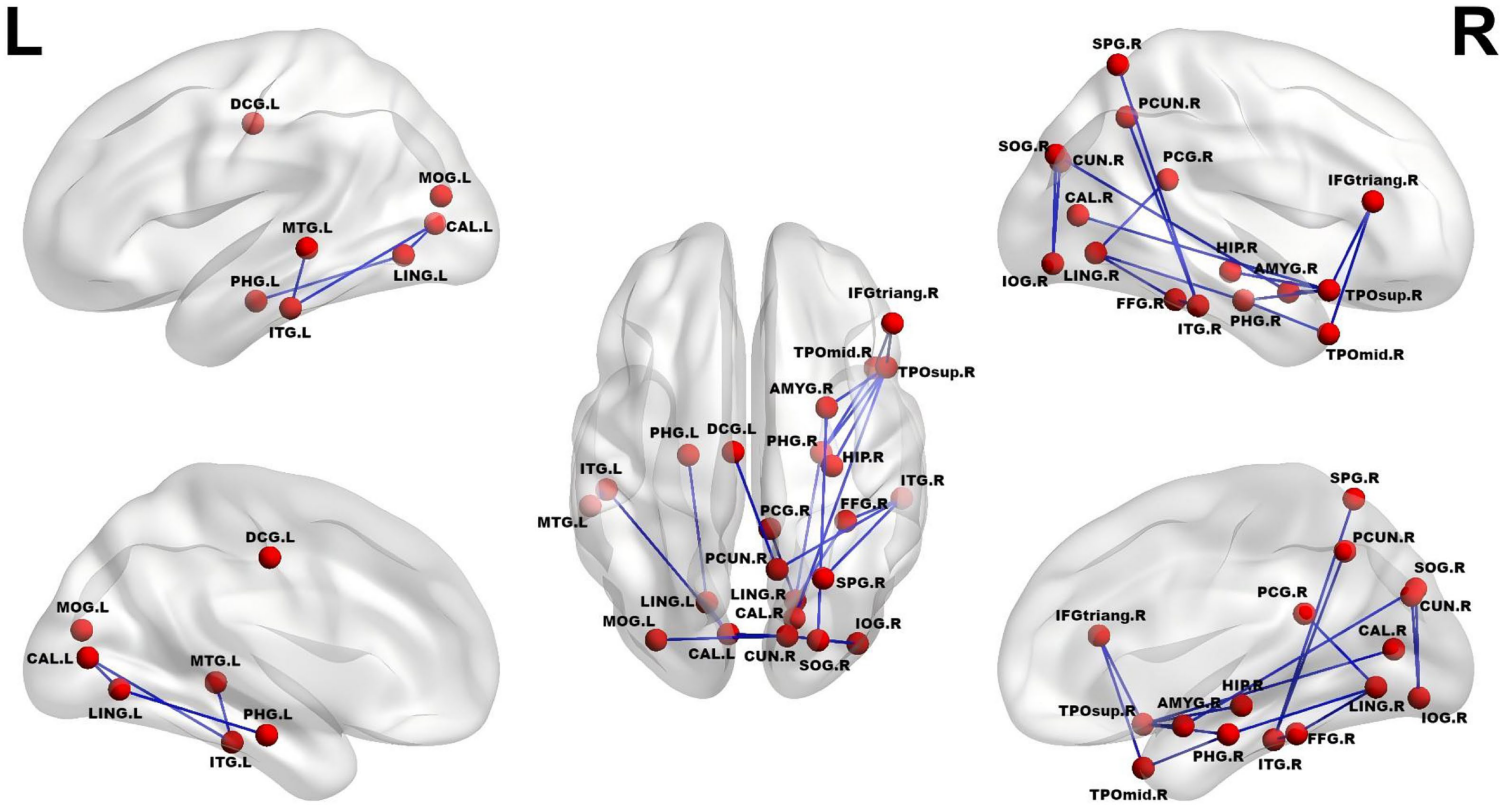
Subnetworks with reduced connection in the MA group compared with the HC group. IFGtriang. R, right inferior frontal gyrus (triangular part); DCG. L, left median cingulate and paracingulate gyrus; PCG. R, right posterior cingulate gyrus; HIP. R, right hippocampus; PHG. L, left parahippocampal gyrus; PHG. R, right parahippocampal gyrus; AMYG. R, right amygdala; CAL. L, left calcarine fissure and surrounding cortex; CAL. R, left and right calcarine fissure and surrounding cortex; CUN. R, right cuneus; LING. L, left lingual gyrus; LING. R, right lingual gyrus; SOG. R, right superior occipital gyrus; MOG. L, left middle occipital gyrus; IOG. R, right inferior occipital gyrus; FFG. R, right fusiform gyrus; SPG. R, right superior parietal gyrus; PCUN. R: right precuneus; TPOsup. R, right temporal pole: superior temporal gyrus); TPOmid. R, right temporal pole: middle temporal gyrus; MTG. L, left middle temporal gyrus; ITG. L, left inferior temporal gyrus; ITG. R, right inferior temporal gyrus.

Compared with the HC group, the MA group exhibited a significantly reduced subnetwork (NBS corrected, *p* < 0.05), which consisted of 24 edges and 23 nodes (see Figure 4). The disrupted nodes within this subnetwork were predominantly clustered into visual processing regions (including the bilateral calcarine fissure, cuneus, lingual gyri, fusiform gyrus, and multiple occipital gyri) and core limbic/paralimbic integration hubs (comprising the right amygdala, hippocampus, bilateral parahippocampal gyri, and cingulate cortex). The remaining nodes encompassed frontoparietal and temporal association cortices, such as the right inferior frontal gyrus (triangular part), right superior parietal gyrus, right precuneus, and lateral temporal regions. However, no significant subnetworks were found between the SA and MA groups (*p* > 0.10, NBS-corrected) or between the SA and HC groups (*p* > 0.10, NBS-corrected).

### 3.5 Network properties and clinical correlations

**Figure 5:**
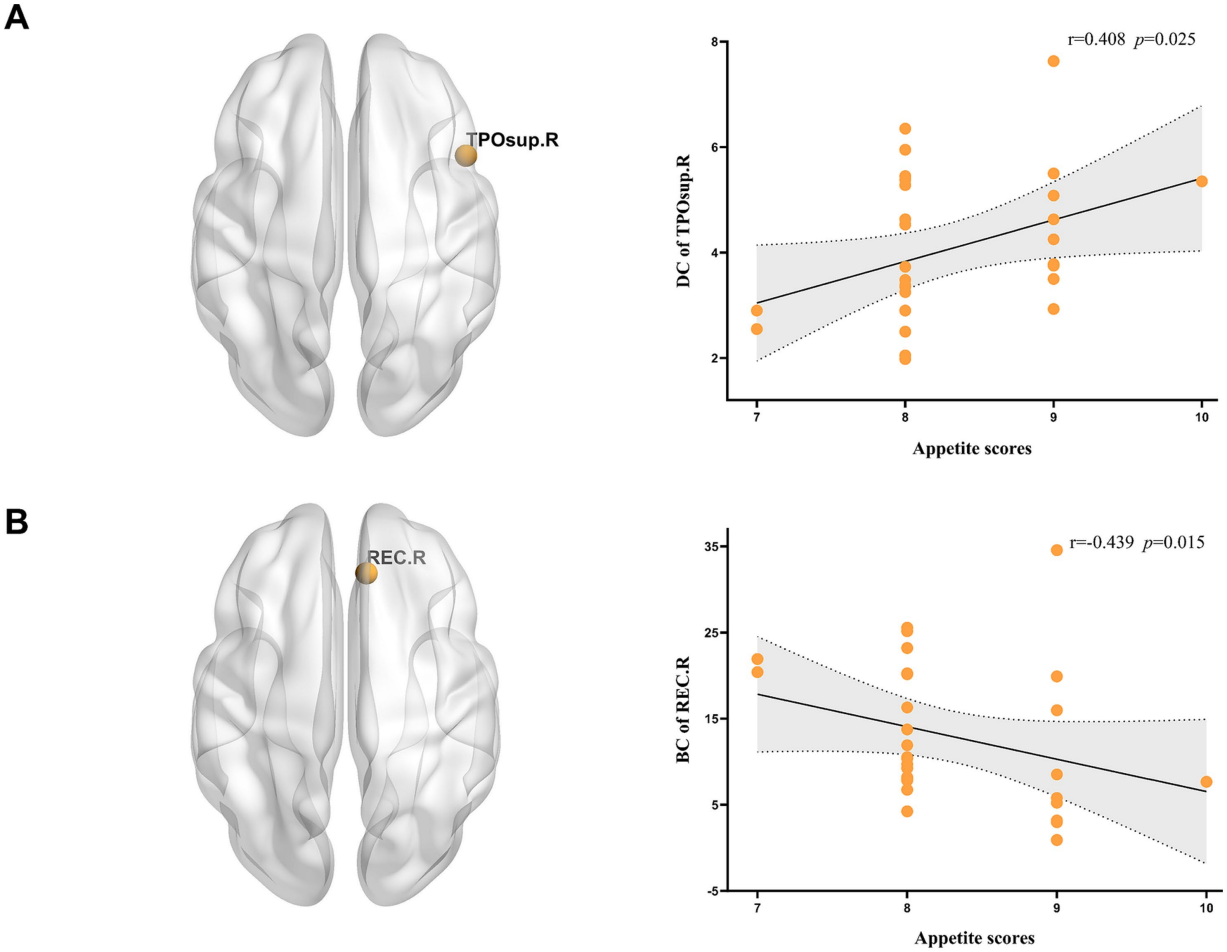
Significant correlations between nodal properties and appetite scores in the SA patients. **(A)** Correlation between DC and VAS of appetite; **(B)** correlation between BC and VAS of appetite. DC, Degree centrality; BC, betweenness centrality; TPOsup. R, right temporal pole: superior temporal gyrus; REC. R, right rectus gyrus; VAS, visual analogue scale.

**Figure 6:**
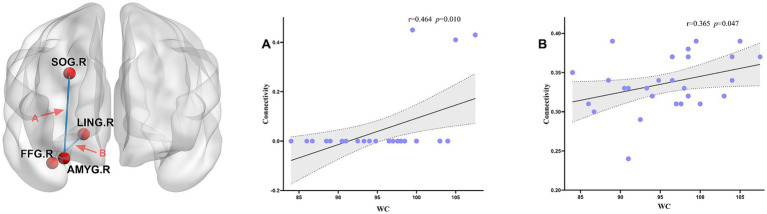
Structural connectivity and clinical correlations analysis. **(A)** Correlation between SOG. R - AMYG. R and WC; **(B)** Correlation between FFG. R - LING. R and WC. AMYG. R, right amygdala; LING. R, right lingual gyrus; SOG. R, right superior occipital gyrus; FFG. R, right fusiform gyrus; WC, waist circumference.

In the SA group, the DC of the TPOsup. R demonstrated a significant positive correlation with appetite scores (*r* = 0.408, *p* = 0.025), whereas the BC of the REC. R showed a negative correlation (*r* = −0.439, *p* = 0.015) (see Figure 5). Structural connectivity analyses for the MA and HC groups demonstrated positive associations with WC (see Figure 6). Specifically, connectivity between the AMYG. R and SOG. R (*p* = 0.01, *r* = 0.464), as well as between the right lingual gyrus (LING. R) and right fusiform gyrus (FFG. R) (*r* = 0.365, *p* = 0.047), was significantly linked to increased abdominal adiposity.

## 4 Discussion

This study is the first to systematically compare the abnormal structural patterns of the whole brain WM network in female with AO exhibiting different appetite subtypes. While the global small-world topology was preserved across all groups, our results revealed that the SA and MA groups exhibited differentiated neural characteristics. Specifically, the SA subtype is associated with alterations in reward-valuation circuits, evidenced by nodal inefficiencies in the right rectus gyrus (a key region of the orbitofrontal cortex) and the amygdala, which were directly correlated with appetite scores. In contrast, the MA subtype is characterized by deficits in metabolic-sensory integration, manifested as a widespread reduction in subnetwork connectivity involving limbic and visual regions (NBS results) that correlated with WC.

### 4.1 Global network robustness and local reorganization

All three groups maintained efficient small-world properties (*γ* > 1, *λ* ≈ 1, *σ* > 1); however, in the global network topology, the small-world parameters γ, λ, σ, and efficiency metrics did not show statistical significance. This result differs from the generally decreased global efficiency in patients with metabolic syndrome (14, 15), suggesting that the structural architecture of the whole-brain WM network in female with AO may be maintained by other compensatory mechanisms to sustain basic communication efficiency (37). Notably, both the SA and MA groups exhibited local WM reorganization. The DC in TPOsup. R was significantly reduced in both the SA and MA groups compared with that in the HC group. This finding suggests that structural impairment in the TPOsup. R is likely a common neuroimaging characteristic of female abdominal obesity. However, we observed a significant positive correlation between the DC of the TPOsup. R and appetite scores exclusively in the SA group. Anatomically, the TPOsup. R is a critical component of the paralimbic system, and recent functional neuroimaging research has further revealed its pivotal role in processing visual food cues and regulating appetite states (38, 39). Based on this, this positive correlation suggests that, acting as a key node for visual cue processing, higher nodal centrality in this region may facilitate a stronger translation of external sensory stimuli into subjective craving. These results suggest that female with AO exhibit different appetite subtypes that may be influenced by key centers rather than global network properties.

### 4.2 Altered nodal topology in reward valuation and salience circuits in the SA subtype

In the SA group, structural network alterations were predominantly characterized by nodal inefficiencies within the prefrontal-limbic circuitry, specifically involving the REC. R, amygdala, and insula. These findings align with the critical roles of the orbitofrontal cortex (OFC) and salience network in the neurobiology of appetite regulation (40–42). Specifically, the reduced BC in the REC. R suggests a diminished influence of this node within the whole-brain network. Anatomically, the rectus gyrus acts as a medial sub-region of the OFC; while early theories emphasized the OFC’s role in inhibitory control, recent evidence underscores its broader function in value-based decision making and the computation of stimulus value (43, 44). Dysfunctional structural connectivity in the OFC has been repeatedly observed in obesity and is thought to impair the ability to update the reward value of food, leading to maladaptive eating despite satiety (45, 46). Our finding of a negative correlation between REC. R centrality and appetite scores supports this interpretation, suggesting that as this critical node for reward valuation becomes less structurally central, the regulation of appetite drive becomes more aberrant. Concurrently, the SA group exhibited reduced NLE in the right amygdala and left insula, which are core components of the salience network responsible for integrating interoceptive signals with emotional relevance to guide behavior (47). Prior neuroimaging studies have identified compromised structural integrity and functional hyper-responsivity in the amygdala-insula circuit among individuals with food addiction or high craving (48–50). In the context of our study, the reduced local efficiency in these nodes implies a disrupted local information transfer, which may hinder the accurate processing of homeostatic signals and enhance the salience attribution to food cues. Therefore, rather than a simple failure of inhibition, the neural signature of the SA subtype appears to involve a disruption in the integration of reward valuation (OFC) and emotional salience (Amygdala/Insula), creating a structural basis for the heightened appetite drive.

### 4.3 Widespread network disruption and metabolic-sensory deficits in the MA subtype

In contrast to the nodal alterations observed in the SA subtype, the MA group exhibited a more widespread disruption of structural connectivity, which may reflect a higher susceptibility to the metabolic burden of obesity. The NBS analysis in the MA group identified a significantly reduced subnetwork comprising 24 edges and 23 nodes. Anatomically, this impaired network predominantly bridges sensory processing areas (e.g., superior occipital, lingual, and fusiform gyri) with central limbic-integration hubs (e.g., amygdala, hippocampus, and parahippocampal gyrus). These regions are integral to the “visual-limbic” circuitry, which is essential for processing external food cues and integrating them with emotional or mnemonic context (51, 52). Structural deficits in this circuit have been linked to altered visual attention to food and impaired regulation of feeding behavior in obesity (53–55). Notably, structural connectivity analyses revealed that the connection strength between the AMYG. R and SOG. R, as well as between the LING. R and FFG. R, was significantly correlated with WC. This significant correlation implies that the severity of abdominal obesity is closely linked to the integrity of structural pathways connecting sensory and limbic systems (56–59). Collectively, these findings suggest that metabolic dysregulation (reflected by elevated WC) may contribute to sensory processing deficits by compromising the structural connectivity required for efficient sensory-limbic integration.

This study has some limitations requiring cautious interpretation. First, the sample size was relatively small (30 cases per group); therefore, a larger sample size may be needed to support the applicability of the results. Second, the cross-sectional design precludes causal inference regarding whether localized network alterations lead to obesity or if they are a result of compensatory remodeling during disease progression; this requires longitudinal tracking to validate temporal relationships. Third, the appetite type relied on VAS, which did not consider the acquisition of endocrine biomarkers (e.g., fasting ghrelin/leptin ratios, HOMA-IR indices) (60, 61). Future multimodal studies should incorporate these biomarkers to provide a more robust biological validation for the heterogeneity between the SA and MA groups. Fourth, regarding data acquisition and processing, the DTI data were acquired with non-isotropic voxels (2 × 2 × 3 mm^3^), which may introduce partial volume effects and limit tractography precision. Similarly, while the AAL-90 template facilitated comparison with prior studies, its relatively coarse resolution may mask regional heterogeneity. Future studies utilizing high-resolution isotropic imaging and fine-grained atlases (e.g., Schaefer-200 or Brainnetome) are thus recommended. Lastly, the lack of functional imaging data highlights the need for multimodal studies (e.g., resting-state fMRI) to confirm the clinical significance of structural network findings.

## 5 Conclusion

In summary, this study provides the first graph-theoretical evidence that females with abdominal obesity exhibiting different appetite phenotypes possess distinct white matter structural network signatures. While the global small-world topology remained resilient across all groups, the underlying regional alterations were subtype-specific. The SA subtype is characterized by aberrant nodal topological properties within reward-valuation and salience circuits, reflecting a disruption in the integration of reward value and emotional salience that underlies heightened appetite. In contrast, the MA subtype is defined by widespread structural disconnectivity within the visual-limbic circuitry, which appears to be a neuroanatomical consequence closely linked to the severity of abdominal adiposity. These findings suggest that “appetite drive” and “metabolic burden” are associated with differential brain network adaptations. Consequently, future clinical interventions should consider these neural heterogeneities, potentially tailoring neuromodulation targets (e.g., enhancing OFC function for SA patients) based on specific appetite subtypes to achieve precision medicine in obesity management.

## Data Availability

The raw data supporting the conclusions of this article will be made available by the authors, without undue reservation.
