# Supplementary material for: Alterations in whole-brain white matter structural network among females with abdominal obesity by appetite subtypes
Source: Front Nutr. 2026 Mar 24;13:1768938. doi: 10.3389/fnut.2026.1768938 (PMC13053284; doi:10.3389/fnut.2026.1768938)
Supplement: Supplementary file 1 [file Table_1.docx]

**Table S1 Results of sensitivity analysis for group differences in nodal topological properties between the SA and MA groups after controlling for BMI and WC.**

| **Brain Region**  **(Node)** | **Metric** | **Group Effect (Adjusted)**  **(F / *p*-value)** | **Robustness Status** |
| --- | --- | --- | --- |
| STG.L | Nodal efficiency | 3.558/0.022^*^ | Robust |
| MOG.R | Nodal local efficiency | 6.013/0.009^*^ | Robust |
| REC.R | Betweenness Centrality | 2.235/0.043^*^ | Robust |

Note: Data were analyzed using General Linear Models (GLM) restricted exclusively to the abdominal obesity cohort (SA and MA groups), with Body Mass Index (BMI) and Waist Circumference (WC) included as covariates. Robustness Status: "Robust" indicates that the statistical significance between SA and MA subtypes was maintained (*p* < 0.05) after adjusting for obesity severity.

Abbreviations: SA, strong appetite; MA, moderate appetite; WC, waist circumference; BMI, body mass index; STG.L, left superior temporal gyrus; MOG.R, right middle occipital gyrus; REC.R, right rectus gyrus.

***** Indicates statistical significance (*p* < 0.05).
